# Long intergenic non-protein coding RNA 00858 functions as a competing endogenous RNA for miR-422a to facilitate the cell growth in non-small cell lung cancer

**DOI:** 10.18632/aging.101171

**Published:** 2017-02-06

**Authors:** Shao-Ping Zhu, Jun-Yu Wang, Xian-Guo Wang, Jin-Ping Zhao

**Affiliations:** ^1^ Department of Cardiothoracic Surgery, ZhongNan Hospital of Wuhan University, 430071 Wuhan, P. R. China; ^2^ Department of Oncology, Hubei Provincial Hospital of Integrated Chinese and Western Medicine, 430015 Wuhan, P. R. China

**Keywords:** long intergenic non-protein coding RNA 00858 (LINC00858), hsa-miRNA-422a (miR-422a), kallikrein-related peptidase 4 (KLK4), non-small cell lung cancer (NSCLC), tumorigenesis

## Abstract

The expression of long non-coding RNAs (lncRNAs) is dysregulated in non-small cell lung cancer (NSCLC). However, the functions and contributions of lncRNAs remain largely unknown. Here, we identified a critical role of long intergenic non-protein coding RNA 00858 (LINC00858) in NSCLC. Ectopic expression of LINC00858 in NSCLC cells promoted cell proliferation and induced cell migration and invasion. Moreover, LINC00858 functioned as a competitive endogenous RNA (ceRNA), effectively becoming sponge for miR-422a and thereby modulating the expression of kallikrein-related peptidase 4 (KLK4). In NSCLC patients, high expression of LINC00858 closely correlated with tumor progression. Thus, targeting the ceRNA network involving LINC00858 may be used as a treatment strategy against NSCLC.

## INTRODUCTION

Non-small cell lung cancer (NSCLC), accounting for 83% of total lung cancer, is a leading cause of all cancer deaths around the world [1]. Main risk factors for NSCLC are cigarette smoke and air pollution [2,3], and the 5-year survival rate for NSCLC patients is lower than 21%, in spite of great efforts to improve treatment options for patients with NSCLC [1]. Increasing evidence reveals that the tumorigenesis and progression for NSCLC are a complicated process referring multiple epigenetic and genetic alterations [4-6]. Hence, upgrades in our knowledge of the molecular alterations (including genetic, epigenetic, protein expression levels) and understanding their functional significance would provide crucial roles on diagnosis, treatment, and prevention for NSCLC.

Long non-coding RNAs (lncRNAs) are a class of non-coding RNAs of more than 200 nucleotides (nt), which are involved in tumor cell proliferation, migration, invasion, apoptosis, angiogenesis, and drug resistance [7-10]. Multiple studies have indicated that lncRNAs, including UCA1 [9], ANRIL [8], XIST [11] and NEAT1 [7] are related to NSCLC tumorigenesis. Nevertheless, the detail biological mechanisms and clinical significance of lncRNAs in the oncogenesis and progression of NSCLC still remains largely unknown.

Long intergenic non-protein coding RNA 00858 (LINC00857, LOC170425, NR_038220), is a kind of lncRNA whose length is 2685 nucleotides and locates in 10q23.1 (http://www.ncbi.nlm.nih.gov/nuccore/NR_038 220.1). Recently, Xu and his colleagues has reported that LINC00858 is highly expressed in lung adenocarcinoma (5.23-fold than normal lung tissues) [12], while until now, the relevance between LINC00858 expression and NSCLC tumorigenesis has not been elaborated yet. Hence, the roles and potential biological mechanisms of LINC00858 on NSCLC still remain to be expounded.

Herein, we are committed to explore the potential molecular mechanism of LINC00858 on NSCLC tumorigenesis. We discovered LINC00858 harbors one conserved miR-422a cognate site as the results of overlap for miRDB (http://mirdb.org/cgi-bin/custom.cgi) and PITA software (http://132.77.150.113/cgi-bin/software.pl?dir=mir07&page=mir07_prediction&id=92e74fcfe41795d7f5b5afd3e80009f7), and then we suggested that LINC00858 would act as a competing endogenous RNA (ceRNA) for miR-422a (MIMAT0001339). Bioinformatics analysis using three database, including microRNA.org, TargetScan, and PicTar, revealed that kallikrein-related peptidase 4 (KLK4), a known oncogene, was an underlying target of miR-422a. Collectively, we validated LINC00858 might be a crucial pro-proliferative regulator to facilitate NSCLC progression by regulation of miR-422a- KLK4 pathway.

## RESULTS

### LINC00858 is up-regulated in NSCLC and its high level implies a poor prognosis

**Figure 1:**
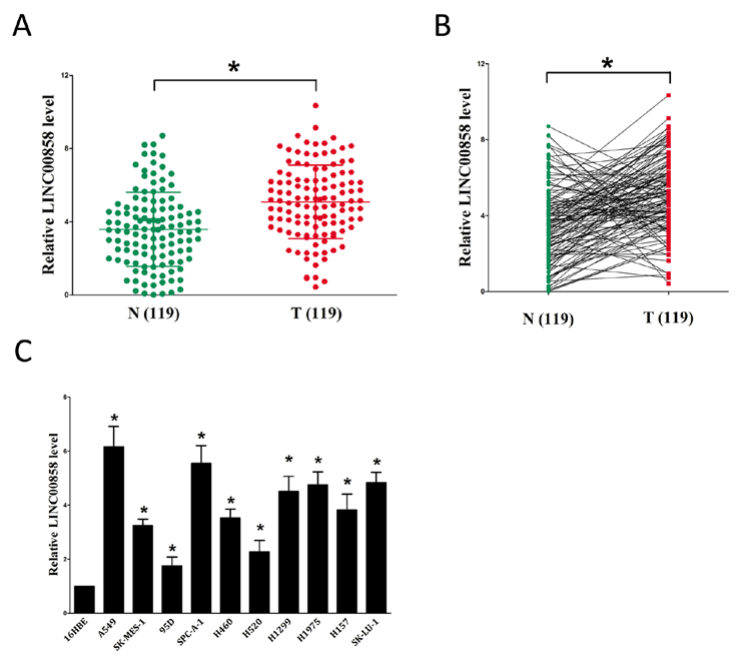
Relative LINC00858 expression in NSCLC tissues and cell lines, and its clinical significance **(A-B)** Relative expression of LINC00858 expression in NSCLC tissues (n = 119) and in paired adjacent normal tissues (n = 119). LINC00858 expression was examined by qPCR and normalized to GAPDH expression (shown as 2^-ΔCT^). **(C)** Relative expression of LINC00858 expression in NSCLC cell lines and normal lung epidermal cell. *P < 0.05. Means ± SEM are shown. Statistical analysis was conducted using student t-test.

To examine if LINC00858 was over-expressed in NSCLC, we first tested LINC00858 expression in human NSCLC tissues and their counterparts by qRT-PCR method. Results revealed that LINC00858 levels were remarkably higher in 119 NSCLC tissues than that of in their counterparts (*P* <0.05) (Fig. 1A-B). Then, we explored LINC00858 expression in NSCLC cell lines, and discovered that LINC00858 was higher expressed in NSCLC cell lines, containing A549, SK-MES-1, H1299, SK-LU-1, H460, H520, 95D, H1975, H157, and SPC-A-1 cell lines, than that of in normal lung epithelial cells, 16HBE (Fig. 1C). LINC00858 are higher expressed in A549 and SPC-A-1 cells among the ten NSCLC cell lines, hence, we selected A549 and SPC-A-1 cells to conduct the forthcoming experiments.

### LINC00858 facilitates tumor NSCLC cell growth *in vitro*

**Figure 2:**
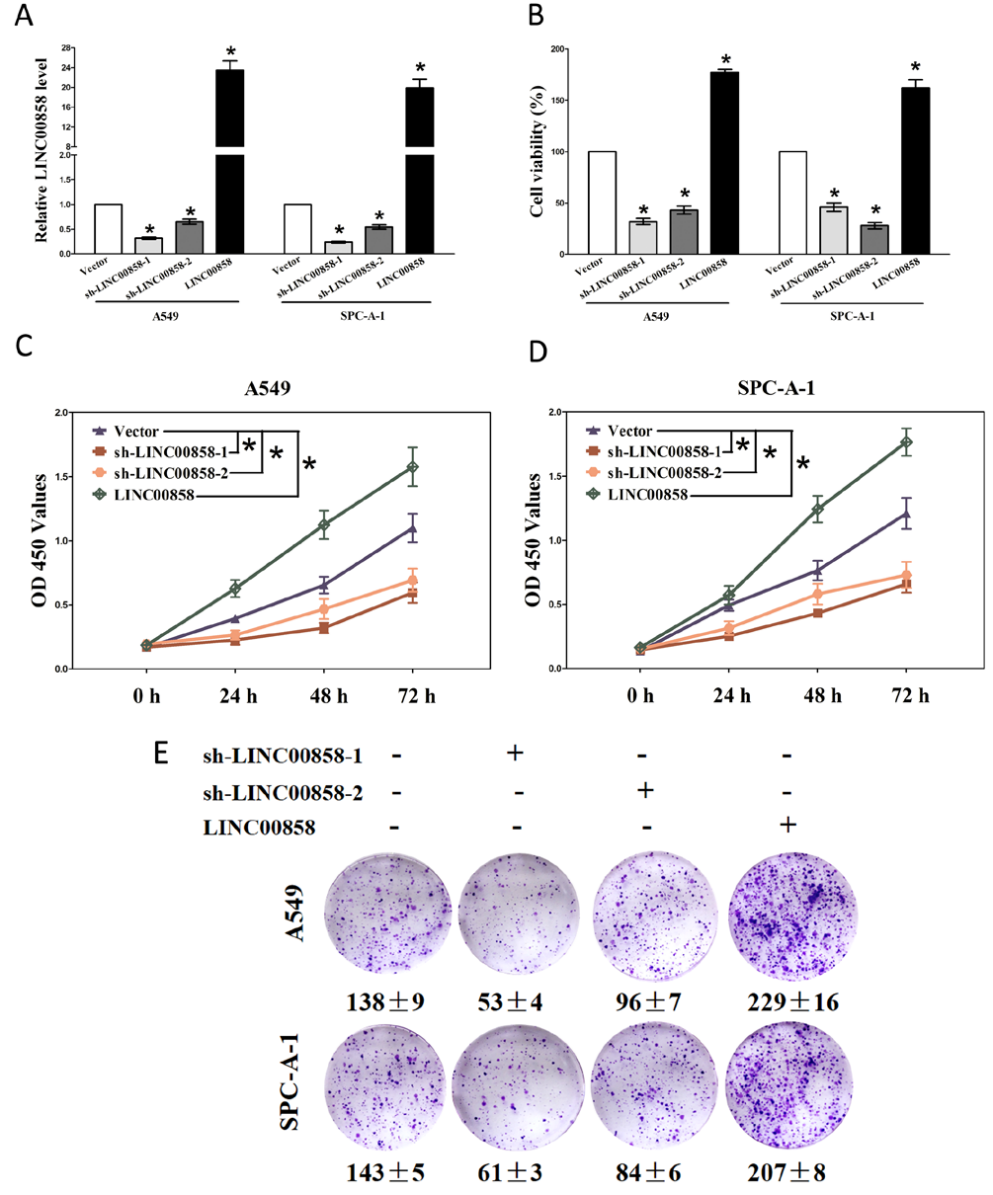
LINC00858 promotes tumor NSCLC cell growth in vitro **(A)** Relative LINC00858 expression after transfection with sh-LINC00858 or pcDNA3.1-CT-GFP-LIN00858. **(B)** Statistical analysis of trypan blue staining. **(C-D)** CCK8 assays of A549 and SPC-A-1 cells after transfection. **(E)** Shown are representative photomicrographs of colony formation assay after transfection for fourteen days. Assays were performed in triplicate. *P < 0.05. Means ± SEM are shown. Statistical analysis was conducted using student t-test.

Having known LINC00858 is high expressed in NSCLC. We then explore its pro-proliferative features and effects on NSCLC. Firstly, we established NSCLC cell lines (A549 and SPC-A-1) with stable over-expressed or knockdown (Using RNAi) for LINC00858. And then, trypan blue staining, CCK8 and colony formation assay were conducted to examine the role of LINC00858 on NSCLC cell growth, and our results revealed knockdown of LINC00858 contributed to a reduction in the cell growth of A549 and SPC-A-1 cells than that of in their blank counterparts (Fig. 2A-E). However, over-expressed LINC00858 facilitated cell growth of A549 and SPC-A-1 cells (Fig. 2A-E), which clearly demonstrated that LINC00858 significantly promoted cell growth in NSCLC cells.

### LINC00858 acts as a ceRNA for miR-422a in NSCLC

**Figure 3:**
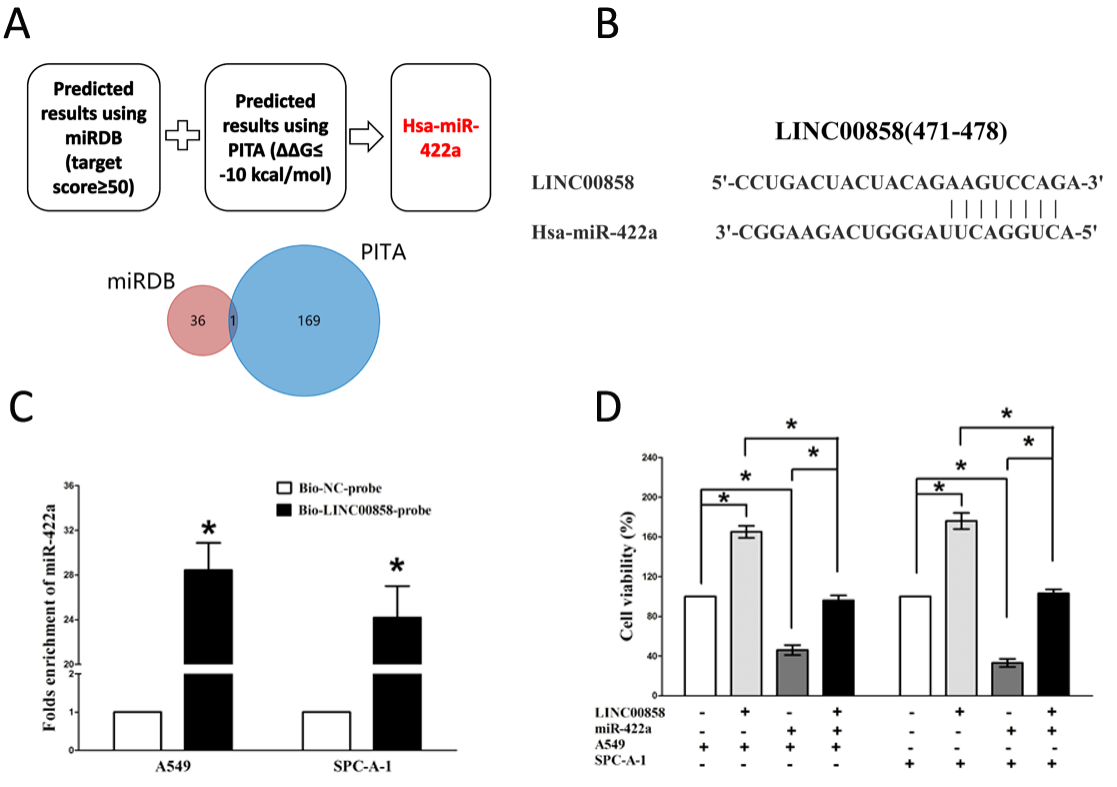
LINC00858 is a direct target of miR-422a **(A)** Screen of the candidate miRNAs that target LINC00858 predicted by miRDB and PITA. **(B)** Sequence alignment of miR-422a with the putative binding sites within the wild-type regions of LINC00858. **(C)** Detection of miR-422a using qRT-PCR in the sample pulled down by biotinylated LINC00858probe. **(D)** Up-regulated miR-422a in A549 and SPC-A-1 cells, which stably over-expressed LINC00858, largely reversed the favorable effects of LINC00858 on cell proliferation. Assays were performed in triplicate. *P< 0.05. Means ± SEM are shown. Statistical analysis was conducted using student t-test.

Recent studies reported lncRNAs could function as molecular sponges or ceRNAs to regulating the biological functions of miRNA. To choose miRNAs interacted with LINC00858, we analyzed the overlap from results of miRDB (http://mirdb.org/cgi-bin/custom.cgi) and PITA software (http://132.77.150.113/cgi-bin/software.pl?dir=mir07&page=mir07_prediction&id=92e74fcfe41795d7f5b5afd3e80009f7) to predict potential miRNAs (results were shown in Table S1 and Table S2). In miRDB, miRNAs with target score≥50 were selected, and in PITA, miRNAs with target score target score ΔΔG≤-10 kcal/mol were selected, then intersection was conducted in the selected miRNAs in miRDB and PITA, and miR-422a was gotten as the candidate miRNA (Table S1 and Table S2). To further verify whether miR-422a was enrichment in LINC00858, we performed a pull-down assay using a biotin-labeled specific LINC00858 probe. And a biotin- labeled NC probe was used as a negative control. qRT-PCR was conducted after precipitate. Results revealed that miR-422a was much richer in precipitate of LINC00858 probe than that of in NC probe (Fig. 3C). These results reveal that miR-422a directly bind to LINC00858 at the recognitive sites. Additionally, we also performed trypan blue staining to explore the interaction between miR-422a and LINC00858 on NSCLC cell growth, and results revealed miR-422a repressed cell growth both in A549 and SPC-A-1 cells, while when co-transfected miR-422a and pcDNA3.1-CT-GFP-LINC00858, the growth-inhibitory role of miR-422a was reversed, while the growth expedited role of LINC00858 was also hampered (Fig. 3D). These data demonstrated that LINC00858 facilitated cell growth through functioning as a ceRNA for miR-422a in NSCLC cell lines.

### LINC00858's pro-proliferative roles are partially via spongeing miR-422a, and then activating KLK4

**Figure 4:**
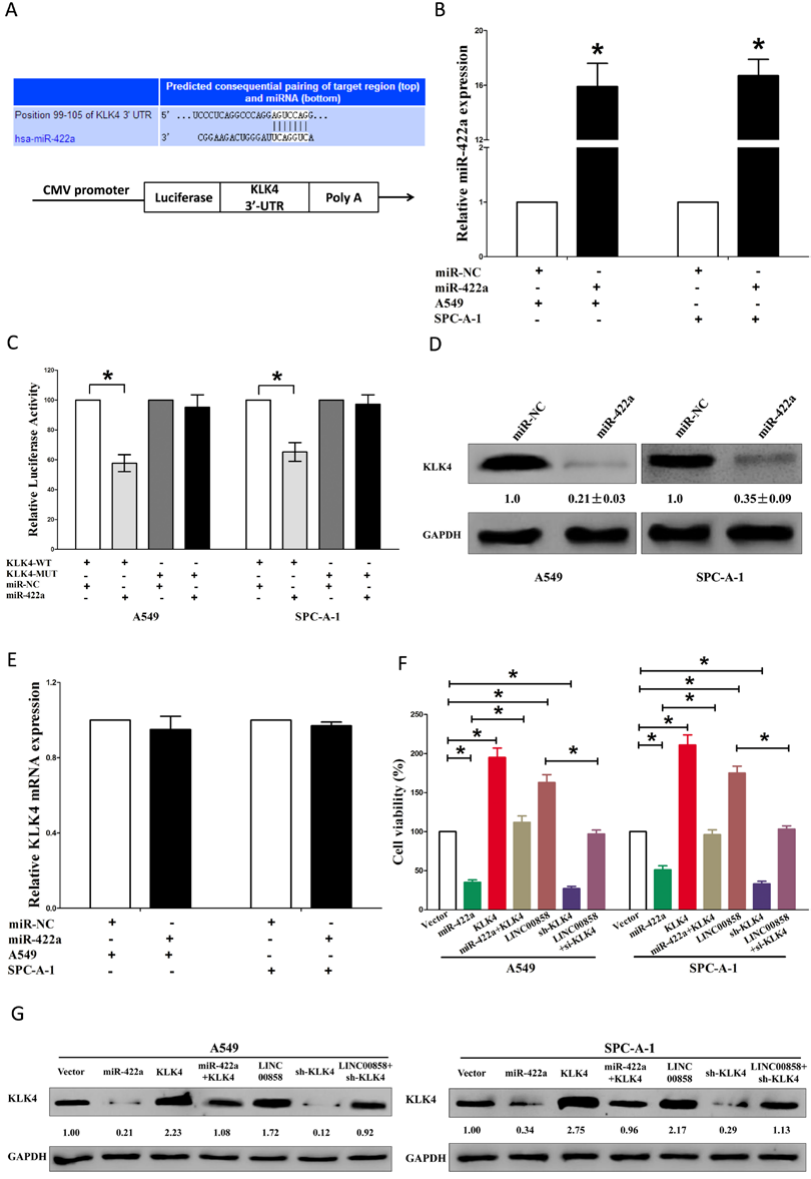
LINC00858's pro-proliferative activity is in part through negative regulation of miRNA-422a, and then activation of KLK4 in NSCLC cells **(A)** The 3'-UTR of KLK4 harbors one miR-422a cognate site. **(B)** Relative miR-422a expression after transfection with miR-NC and miR-422a. **(C)** Relative luciferase activity of reporter plasmids carrying wild-type or mutant KLK4 3'-UTR in A549 and SPC-A-1 cells co-transfected with miR-NC or miR-422a. **(D)** Relative KLK4 protein expression after transfection with miR-NC and miR-422a. **(E)** Relative KLK4 mRNA expression after transfection with miR-NC and miR-422a. **(F)** Statistical analysis of trypan blue staining. **(G)** Protein expression of KLK4 in Vector, miR-422a, pcDNA3.1-CT-GFP-KLK4, miR-422a plus pcDNA3.1-CT-GFP-KLK4, pcDNA3.1-CT-GFP-LINC00858, sh-KLK4, or pcDNA3.1-CT-GFP-LINC00858 +sh-KLK4 treated A549 and SPC-A-1 cells. Assays were performed in triplicate. *P < 0.05. Means ± SEM are shown. Statistical analysis was conducted using student One-Way ANOVA test.

**Figure 5:**
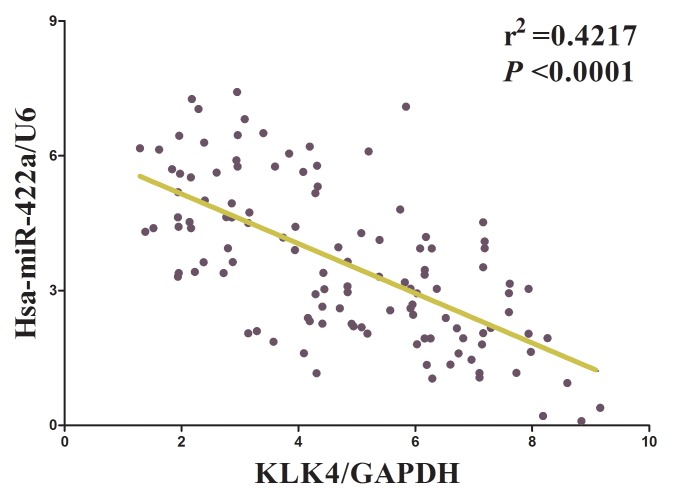
The relationship between KLK4 mRNA expression and miR-422a expression in non-small cell lung cancer

Having confirmed LINC00858 could inversely regulate miR-422a's inhibitory role on cell growth in NSCLC, we then explore its functional effects. MiRNAs play crucial role on tumor progression [13-20]. To explore the role of miR-422a on NSCLC, we screen miRanda, Targetscan, and PicTar to select underlying predicted targets for miR-422a. We seek out top 100 potential targets (Table S3), and among them, we selected a well-known oncogene, kallikrein-related peptidase 4 (KLK4), which was up-regulated in numerous malignancies, as a potential target (Fig. 4A). Then, we conducted luciferase reporter assays to identify if KLK4 expression was really affected by miR-422a, and results demonstrated that transfection with miR-422a significantly up-regulated the miR-422a expression in A549 and SPC-A-1 cells(Fig. 4B), and miR-422a inhibited luciferase activity in both A549 cells and SPC-A-1 cells that transfected with a wide type (WT) KLK4 3′-UTR reporter plasmid, but no significant inhibition was observed in that of transfected with a mutant (MUT) KLK4 3′-UTR reporter plasmid (Fig. 4C). Moreover, miR-422a decreased the protein expression but had no influence on the mRNA expression for KLK4 in A549 and SPC-A-1 cells (Fig. 4D-E). We then explored the mechanism of miR-422a on NSCLC cell growth. Results of Trypan blue staining revealed miR-422a treatment repressed cell growth and pcDNA3.1-CT-GFP-KLK4 (with a full 3′-UTR mRNA for KLK4) treatment increased cell growth in A549 and SPC-A-1 cells (Fig. 4F). Nevertheless, when treated A549 and SPC-A-1 cells with miR-422a plus pcDNA3.1-CT-GFP-KLK4, the advantageous role of KLK4 on cell growth was repressed by miR-422a, and the growth-inhibitory role of effect of miR-422a was reversed by KLK4 over-expression (Fig. 4F). These data revealed that miR-422a repressed cell growth by directly targeting 3′-UTR of KLK4 mRNA. Additionally, pcDNA3.1-CT-GFP-LINC00858 treatment reversed the growth inhibitory effect of sh-KLK4 in A549 and SPC-A-1 cells (Fig. 4F), which demonstrated that LINC00858 facilitated cell growth partially through up regulation of KLK4. Furthermore, we next explored the role of LINC00858 and miR-422a on the protein expression of KLK4. Results demonstrated that both miR-422a and sh-KLK4 treatment suppressed protein expression of KLK4, while both pcDNA3.1-CT-GFP-LINC00858 and pcDNA3.1-CT-GFP-KLK4 treatment markedly enhanced protein expression of KLK4 in A549 and SPC-A-1 cells (Fig. 4G), separately. Nevertheless, when treated A549 and SPC-A-1 cells with pcDNA3.1-CT-GFP-LINC00858 plus sh-KLK4, the beneficial role of LINC00858 on protein expression of KLK4 was repressed by knockdown of KLK4, and the negative effect of sh-KLK4 was attenuated by over-expression of LINC00858 (Fig. 4G). Additionally, our results also demonstrated the mRNA of KLK4 expression had no significant correction with the expression of miR-422a in NSCLC samples (r^2^ =0.0171, *P* =0.1559), and miR-422a did not affect the mRNA of KLK4 in NSCLC cell lines. While over-expressed miR-422a markedly suppressed the protein expression of KLK4, which demonstrated that miR-422a regulated the KLK4 at post-transcription level (Fig. 5). Our data identify that miR-422a directly targets 3′-UTR of KLK4 mRNA, and reveal that LINC00858's pro-proliferative effects are large in part by negative regulating miR-422a, and then activation of KLK4.

## DISCUSSION

Increasing evidence has revealed lncRNAs might play a crucial role in tumorigenesis, and contributes to a diverse of biological functions in human cancers [8-10]. Up to date, the effects of a handle of lncRNAs in NSCLC tumorigenesis and progression are needed fully elucidated. Recent studies suggest that several lncRNAs are dysregulated in multiple cancers, including NSCLC [21-25]. One of these is nuclear-enriched abundant transcript 1 (NEAT1), which is a highly conserved nuclear lncRNA and a predictive marker for metastasis in lung cancer [22]. In the current study, we discovered that the lncRNA LINC00858 was up-regulated in NSCLC, and exerted a pro-proliferative role. We also discovered that miR-422a is the target of LINC00858. LINC00858 exerted its pro-proliferative role on NSCLC in large part due to its effect to act as a ceRNA for miR-422a, and subsequently activating the KLK4 signaling pathway. Hence, these findings indicate that LINC00858 may function as an oncogene in the modulation of NSCLC progression.

To our knowledge, our present work is the first one to direct investigate of the association between LINC00858 expression and NSCLC. In this study, we discovered LINC00858 was markedly higher expressed in NSCLC tissues than that of in their counterparts. Additionally, over-expressing LINC00858 markedly facilitated NSCLC cell growth in vitro, while silencing LINC00858 suppressed it. Collectively, our data imply that LINC00858 may function as an oncogene and be favorable to NSCLC tumorigenesis and progression.

Despite LINC00858 has been implied to function as an oncogene, the potential mechanisms by which LINC00858-induced gene expression involved in oncogenesis still remains to be further expounded. Herein, we are committed to excavate a potential biological molecular mechanism of LINC00858 on NSCLC progression. Increasing evidence confirmed that lncRNAs are key regulators in numerous processes in cells via functioning as ceRNAs to control microRNAs [26]. A handle of lncRNAs have been assessed, such as GAS5 [27, 28], NEAT1 [22], XIST [29], and CCAT1 [30]. Herein, we explored the role of LINC00858 in NSCLC and found that LINC00858 acted as ceRNA to bind with miR-422a and accommodate its function. It has been reported that miR-422a acted as a potential tumor suppressor was an independent prognostic factor in colorectal cancer [31], and promoted loco-regional recurrence by targeting NT5E/CD73 in head and neck squamous cell carcinoma [32], but its role and potential mechanism on NSCLC needed to be warranted. Herein, we found that miR-422a inhibited growth in NSCLC cell lines, and biotin-avidin pull-down assay revealed LINC00858 could pull down miR-422a. Furthermore, our data also demonstrated that miR-422a could inverse the advantageous effects of LINC00858 on cell growth in NSCLC, suggesting LINC00858 exerted an pro-proliferative role on NSCLC progression in large part via repressing miR-422a.

Having revealed the critical effect of miR-422a on repressing NSCLC progression, we searched for the potential mechanisms participated in its function. MiR-422a could regulate multiple targeted genes. It has been reported that miR-422a targeted NT5E/CD73 and promoted loco-regional recurrence in head and neck squamous cell carcinoma [32]. But among all of the predicted target genes for miR-422a, we discovered KLK4 functioned as a critical effector of miR-422a. KLK4 is key regulator on the proliferation of several tumor cell lines [33-35], and is associated with higher risk of NSCLC as determined by univariate analysis and confirmed by multivariate analysis [36]. We confirmed that over-expressed KLK4 promoted cell proliferation in NSCLC cell lines, while silencing KLK4 expression suppressed it. Moreover, we predicted KLK4 as a direct target of miR-422a at its 3′-UTR mRNA by bioinformatics, which was confirmed by luciferase reporter assays. Our results also demonstrated that miR-422a regulated the KLK4 at post-transcription level. Additionally, our data also revealed miR-422a acted as a tumor suppressor on NSCLC by directly targeting KLK4. Furthermore, our data also revealed that LINC00858's pro-proliferative roles are partially via sponging miR-422a, and then activating KLK4.

To our knowledge, we first proved that LINC00858 exerts its pro-proliferative effect in part through sponging miR-422a and then up-regulating and activating of KLK4. Our present study supplied an unequivocal evidence for crucial pro-proliferative effects of LINC00858 in NSCLC, and endorsed the conception that LINC00858 might be an alternative pro-proliferative factor for NSCLC. Taken together, our data provide the evidence that targeting LINC00858-miR-422a-KLK4 axis might be an alternative therapeutic strategy, which will help furnish an essential implication for treatment and diagnosis of NSCLC.

## MATERIALS AND METHODS

### Ethical statement

For the analyzed tissue specimens, all patients gave informed consent to use excess pathological specimens for research purposes. The protocols employed in this Subjects Committee. The use of human tissues was approved by the institutional review board of the Wuhan University and conformed to the Helsinki Declaration and to the local legislation. Patients offering samples for the study signed informed consent forms.

### Tissue collection

119 cases of NSCLC tumor tissue samples were obtained from patients who were diagnosed with primary NSCLC at ZhongNan Hospital of Wuhan University (Wuhan, China) from 2009-2014. The use of tissues for this study has been approved by the ethics committee of ZhongNan Hospital of Wuhan University. Before using these clinical materials for research purposes, all the patients signed the informed consent. None of these patients received any pre-operative chemotherapy or radiotherapy.

### Cell lines and plasmids

Ten NSCLC cell lines (A549, SK-MES-1, H1299, 95D, H460, H520, H1975, H157, SK-LU-1, and SPC-A-1) and the 16HBE cell lines were purchased from the Institute of Biochemistry and Cell Biology of the Chinese Academy of Sciences (Shanghai, China). Cells were cultured in RPMI 1640 (Gibco, Grand Island, NY, USA) medium supplemented with 10% fetal bovine serum (10% FBS), 100 U/ml penicillin, and 100 mg/ml streptomycin (Gibco) in humidified air at 37°C with 5% CO2. Plasmid pcDNA3.1-CT-GFP-KLK4 (NM_004917, with full length of 3′-UTR) and pcDNA3.1-CT-GFP-LINC00858 were prepared by ourselves. RNAi sequence: **KLK4**: sh-1: CACCGCTGCAGCCAAATCATAAACGCGAACGTTTATGATTTGGCTGCAGC; sh-2: CACCGGTCTGCAGTAAGCTCTATGACGAATCATAGAGCTTACTGCAGACC. **LINC00858**: sh-1: CACCGGACTTCATGGTTCAGCATCACGAATGATGCTGAACCATGAAGTCC; sh-2: CACCGCAGCAGCCAGATGAGAAATTCGAAAATTTCTCATCTGGCTGCTGC.

### Cell transfection and stable cell lines

Cells were transfected with DNA plasmids using transfast transfection reagent LipofectamineR 2000 (Invitrogen) according to manufacturer's instructions [37]. For screening stable cell lines, forty-eight hours after transfection, cells were plated in the selective medium containing G418 (1000–2000 μg/ml, Invitrogen, Ltd., U.K) for the next 4 weeks or so, and the selective media were replaced every 3 days.

### Western blot analysis

Western blot was performed using the protocol described previously [38-40]. Briefly, the logarithmically growing cells were washed twice with ice-cold phosphate-buffered saline (PBS, Hyclone) and lysed in a RIPA lysis buffer. Cells lysates were centrifuged at 12,000 g for 20 minutes at 4°C after sonication on ice, and the supernatant were separated. After being boiled for 5–10 minutes in the presence of 2-mercaptoethanol, samples containing cells proteins were separated on a 10% sodium dodecyl sulfate-polyacrylamide gel electrophoresis (SDS-PAGE) and transferred onto a nitrocellulose membranes. Then blocked in 10% dry milk-TBST (20 mM Tris-HCl [PH 7.6], 127 mM NaCl, 0.1% Tween 20) for 1 h at 37°C. Following three washes in Tris-HCl pH 7.5 with 0.1% Tween 20, the blots were incubated with 0.2 μg/ml of antibody (appropriate dilution) overnight at 4°C. Following three washes, membranes were then incubated with secondary antibody for 60 min at 37°C or 4°C overnight in TBST. Signals were visualized by ECL. The following primary antibodies were used: rabbit anti-KLK4 (Santa Cruz, USA), rabbit anti-GAPDH (Santa Cruz, USA).

### qRT-PCR

RNA isolation and qRT-PCR was performed as described previously [41,42]. GADPH and U6 were used as endogenous controls. In addition, melting curves were used to evaluate non-specific amplification. The relative expression level was calculated using the 2^-ΔΔCt^ method. The primer sequences used in this study are as follows: human KLK4: sense: 5'- CCGCACACTGTTTCCAGAAC-3', antisense: 5'- GTTAGCGAGCAAGGGTCTGT-3', product length is 140; human LINC00858: sense: 5'- CCCAGCTCCTTACACACGTT-3', antisense: 5'- TTCAGAGGCCTGCATCACTG-3'; human GAPDH: sense: 5'-CTCTGCTCCTCCTGTTCGAC-3', antisense: 5'-ACCAAATCCGTTGACTCCGA -3'. The formula and its derivations were obtained from the ABI Prism 7300 sequence detection system user guide. Statistical analysis was performed on the fold change.

### Colony formation assay

Colony formation assay was conducted as described previously [41,42].

### Luciferase Reporter Assays

Luciferase reporter assays was conducted as described previously [4].

### CCK8 Assay

Cell growth was measured using the cell proliferation reagent WST-8 (Roche Biochemicals, Mannheim, Germany). After plating cells in 96-well microtiter plates (Corning Costar, Corning, NY) at 1.0× 10^3^ /well, 10 μL of CCK8 was added to each well at the time of harvest, according to the manufacturer's instructions. One hour after adding CCK8, cellular viability was determined by measuring the absorbance of the converted dye at 450 nm.

### Trypan blue staining

Cell viability was assessed using the trypan blue (Lonza, Basel, Switzerland) exclusion method. The A549 and SPC-A-1 cells were seeded in 24-well culture plates at a density of 3 × 10^5^ cells per well, and the cells were then transfected with Vector, miR-422a, pcDNA3.1-CT-GFP-KLK4, miR-422a plus pcDNA3.1-CT-GFP-KLK4, pcDNA3.1-CT-GFP-LINC00858, sh-LINC00858, or pcDNA3.1-CT-GFP-LINC00858 plus sh-LINC00858. Each cell suspension was mixed with an equal volume of 0.4% trypan blue solution, and the living cells were quantified using a hemocytometer. The cells were also counted using a microscope. The data are representative of three independent experiments performed on different days.

### RNA pull-down assays

LINC00858 transcripts were transcribed using T7 RNA polymerase (Ambio life) *in vitro*, then by using the RNeasy Plus Mini Kit (Qiagen) and treated with DNase I (Qiagen). Purified RNAs were biotin-labeled with the Biotin RNA Labeling Mix (Ambio life). Positive control, negative control and Biotinylated RNAs were mixed and incubated with A549 and SPC-A-1 cell lysates. Then, magnetic beads were added to each binding reaction, and incubated at room temperature. Finally, the beads were washed, and the eluted proteins were detected by western blot analysis.

### Statistical analysis

Statistical analyses were carried out using SPSS 23.0 statistical analysis software (SPSS Inc., Chicago, IL, USA). Two independent sample t-test or One-Way Analysis of Variance (ANOVA) was performed using SPSS 20.0 software to assess significant differences in measured variables among groups. All data are presented as mean ± standard error of mean (SEM) for three independent experiments. A *P* value < 0.05 was considered statistically significant.

## SUPPLEMENTAL MATERIAL


