# Supplementary material for: Long intergenic non-protein coding RNA 00858 functions as a competing endogenous RNA for miR-422a to facilitate the cell growth in non-small cell lung cancer
Source: Aging (Albany NY). 2017 Feb 6;9(2):475–85. doi: 10.18632/aging.101171 (PMC5361675; doi:10.18632/aging.101171)
Supplement: Supplementary file 1 [file aging-09-475-s001.pdf]

SUPPLEMENTARY MATERIAL

Supplementary Table S1. Predicted results using miRDB (target score≥50)

Target Detail	Target Rank	Target Score	miRNA Name	Gene Symbol
	1	81	hsa-miR-3182	submission
	2	78	hsa-miR-4419a	submission
	3	68	hsa-miR-6513-3p	submission
	4	67	hsa-miR-4753-3p	submission
	5	66	hsa-miR-4291	submission
	6	63	hsa-miR-6819-5p	submission
	7	63	hsa-miR-4633-3p	submission
	8	60	hsa-miR-1227-5p	submission
	9	59	hsa-miR-6124	submission
	10	57	hsa-miR-7973	submission
	11	56	hsa-miR-6842-3p	submission
	12	55	hsa-miR-6756-3p	submission
	13	55	hsa-miR-3127-3p	submission
	14	55	hsa-miR-4769-5p	submission
	15	55	hsa-miR-4654	submission
	16	55	hsa-miR-4516	submission
	17	55	hsa-miR-4434	submission
	18	53	hsa-miR-6818-3p	submission
	19	53	hsa-miR-6872-3p	submission
	20	52	hsa-miR-6882-3p	submission
	21	52	hsa-miR-153-3p	submission
	22	52	hsa-miR-6809-3p	submission
	23	51	hsa-miR-4778-3p	submission
	24	51	hsa-miR-6776-3p	submission
	25	51	hsa-miR-653-5p	submission
	26	50	hsa-miR-6736-3p	submission
	27	50	hsa-miR-6844	submission
	28	50	hsa-miR-422a	submission
	29	50	hsa-miR-378i	submission
	30	50	hsa-miR-378h	submission
	31	50	hsa-miR-378f	submission
	32	50	hsa-miR-378e	submission
	33	50	hsa-miR-378d	submission
	34	50	hsa-miR-378c	submission
	35	50	hsa-miR-378b	submission
	36	50	hsa-miR-378a-3p	submission
	37	50	hsa-miR-3152-3p	submission

Supplementary Table S2. Predicted results using PITA (target score≤-10 kcal/mol) - Supplementary File 1

Supplementary Table S3. Top 100 potential targets of miR-422a - Supplementary File 2
